# Exploiting the Immunomodulatory Properties of Chemotherapeutic Drugs to Improve the Success of Cancer Immunotherapy

**DOI:** 10.3389/fimmu.2015.00516

**Published:** 2015-10-07

**Authors:** Kelly Kersten, Camilla Salvagno, Karin E. de Visser

**Affiliations:** ^1^Division of Immunology, Netherlands Cancer Institute, Amsterdam, Netherlands

**Keywords:** cancer immunotherapy, immune checkpoint blockade, chemotherapy, tumor microenvironment, immunosuppression, anti-tumor immunity

## Abstract

Cancer immunotherapy is gaining momentum in the clinic. The current challenge is to understand why a proportion of cancer patients do not respond to cancer immunotherapy, and how this can be translated into the rational design of combinatorial cancer immunotherapy strategies aimed at maximizing success of immunotherapy. Here, we discuss how tumors orchestrate an immunosuppressive microenvironment, which contributes to their escape from immune attack. Relieving the immunosuppressive networks in cancer patients is an attractive strategy to extend the clinical success of cancer immunotherapy. Since the clinical availability of drugs specifically targeting immunosuppressive cells or mediators is still limited, an alternative strategy is to use conventional chemotherapy drugs with immunomodulatory properties to improve cancer immunotherapy. We summarize the preclinical and clinical studies that illustrate how the anti-tumor T cell response can be enhanced by chemotherapy-induced relief of immunosuppressive networks. Treatment strategies aimed at combining chemotherapy-induced relief of immunosuppression and T cell-boosting checkpoint inhibitors provide an attractive and clinically feasible approach to overcome intrinsic and acquired resistance to cancer immunotherapy, and to extend the clinical success of cancer immunotherapy.

## Introduction

Cancer immunotherapy – harnessing the patient’s immune system against cancer – is currently gaining momentum in the clinic. Clinical trials with immune checkpoint inhibitors show remarkable success in patients with advanced metastatic melanoma, non-small cell lung cancer, renal cancer, bladder cancer, and Hodgkin’s lymphoma (1–6). As a result, the journal *Science* proclaimed cancer immunotherapy as the breakthrough of 2013 (7). Furthermore, these encouraging results led to FDA approval of the immune checkpoint inhibitors ipilimumab (anti-CTLA-4), nivolumab, and pembrolizumab (anti-PD-1) in the past few years. Although cancer immunotherapy was proclaimed a breakthrough, a significant proportion of cancer patients do not show clinical benefit. There are various cancer cell-intrinsic and cancer cell-extrinsic processes that regulate intrinsic or acquired resistance to cancer immunotherapy. Cancer cell-intrinsic characteristics like the mutational load have been reported to affect responsiveness to immunotherapy (8, 9). In terms of cancer cell-extrinsic processes, tumors exploit different strategies to induce immune escape by hampering the recruitment and activation of effector T cells, and by creating a local immunosuppressive environment through recruitment of suppressive myeloid and regulatory T cells that dampen T cell effector functions. Which of these immune escape mechanisms are active in a certain tumor depends on the tumor type, tumor stage, and therapy history. A deeper understanding of the molecular mechanisms underlying these processes will contribute to the identification of biomarkers that can predict therapeutic efficacy of immunotherapy and to the design of combinatorial strategies aimed at maximizing the success of immunotherapy.

In this review, we discuss how tumor-induced immunosuppressive networks counteract efficacious anti-tumor immune responses, and how disruption of these networks can increase the anti-cancer efficacy of cancer immunotherapy with immune checkpoint inhibitors. Development and clinical testing of novel drugs specifically targeting immunosuppressive networks are ongoing and preliminary results are promising (10). An alternative strategy to relieve tumor-induced immunosuppressive states is to use conventional, and more easily accessible, anti-cancer treatment strategies with known immunomodulatory properties, such as chemotherapy, radiotherapy, and targeted therapy (11–15). Here, we focus on the immunomodulatory properties of conventional chemotherapy, and how these properties can be exploited to improve the anti-cancer efficacy of immune checkpoint inhibitors.

## Cancer Immunotherapy: Opportunities and Challenges

### Tumor-induced mechanisms of immune escape

**Figure 1:**
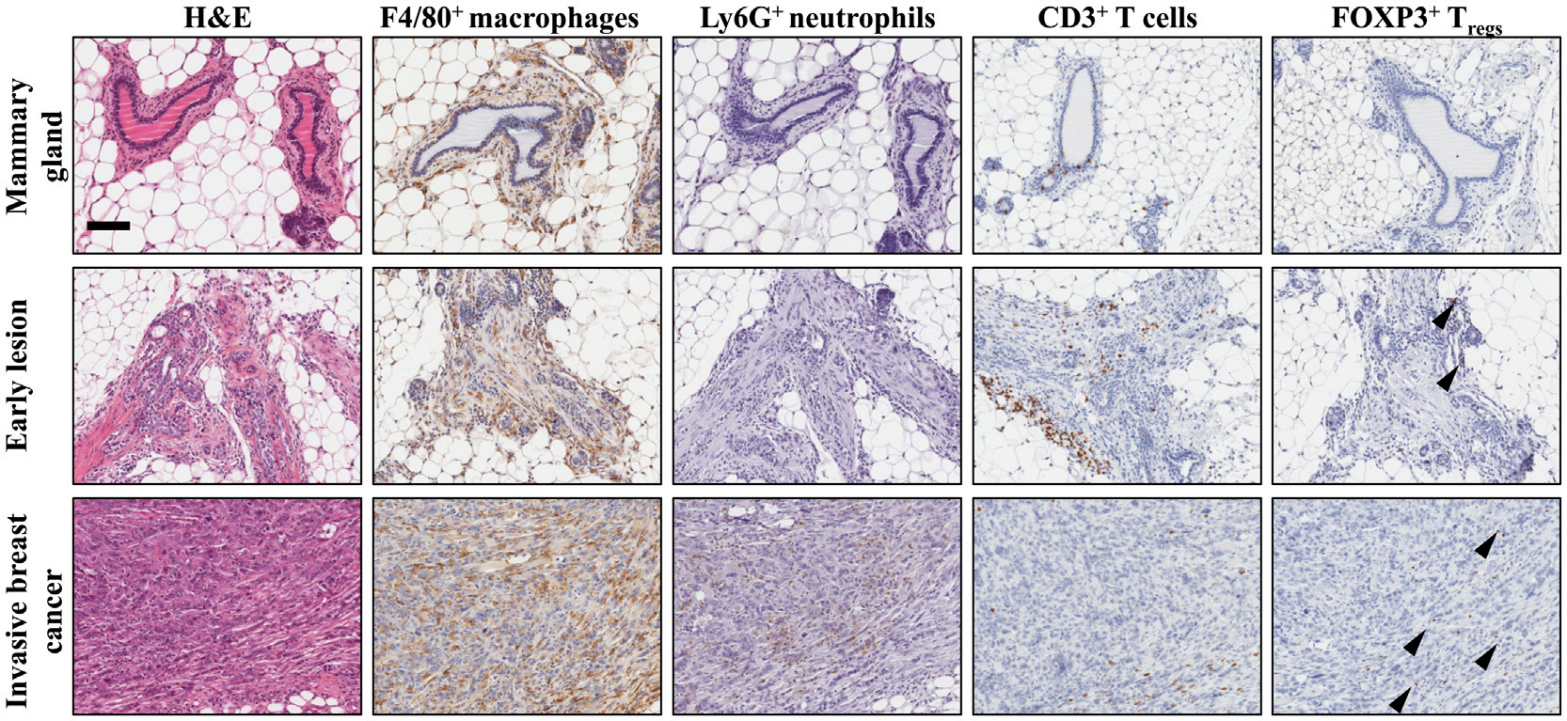
**Establishment of the immune microenvironment during breast cancer progression in a conditional mouse model for mammary tumorigenesis**. Female *K14Cre;Cdh1^F/F^;Trp53^F/F^* mice develop *de novo* invasive mammary tumors that closely resemble human invasive lobular carcinoma (19). Immunohistochemical staining on mammary tissue from *K14Cre;Cdh1^F/F^;Trp53^F/F^* mice obtained during different stages of mammary tumor progression. From top to bottom are represented wild-type mammary gland (top), early lesion (middle), established mammary tumor (bottom). From left to right, identification of different immune cell populations by H&E, F4/80 (macrophages), Ly6G (neutrophils), CD3 (total T cells), and FOXP3 (regulatory T cells) staining showing the dynamics of the tumor microenvironment. Arrowheads indicate FOXP3^+^ nuclei. Scale bar 100 μm.

**Figure 2:**
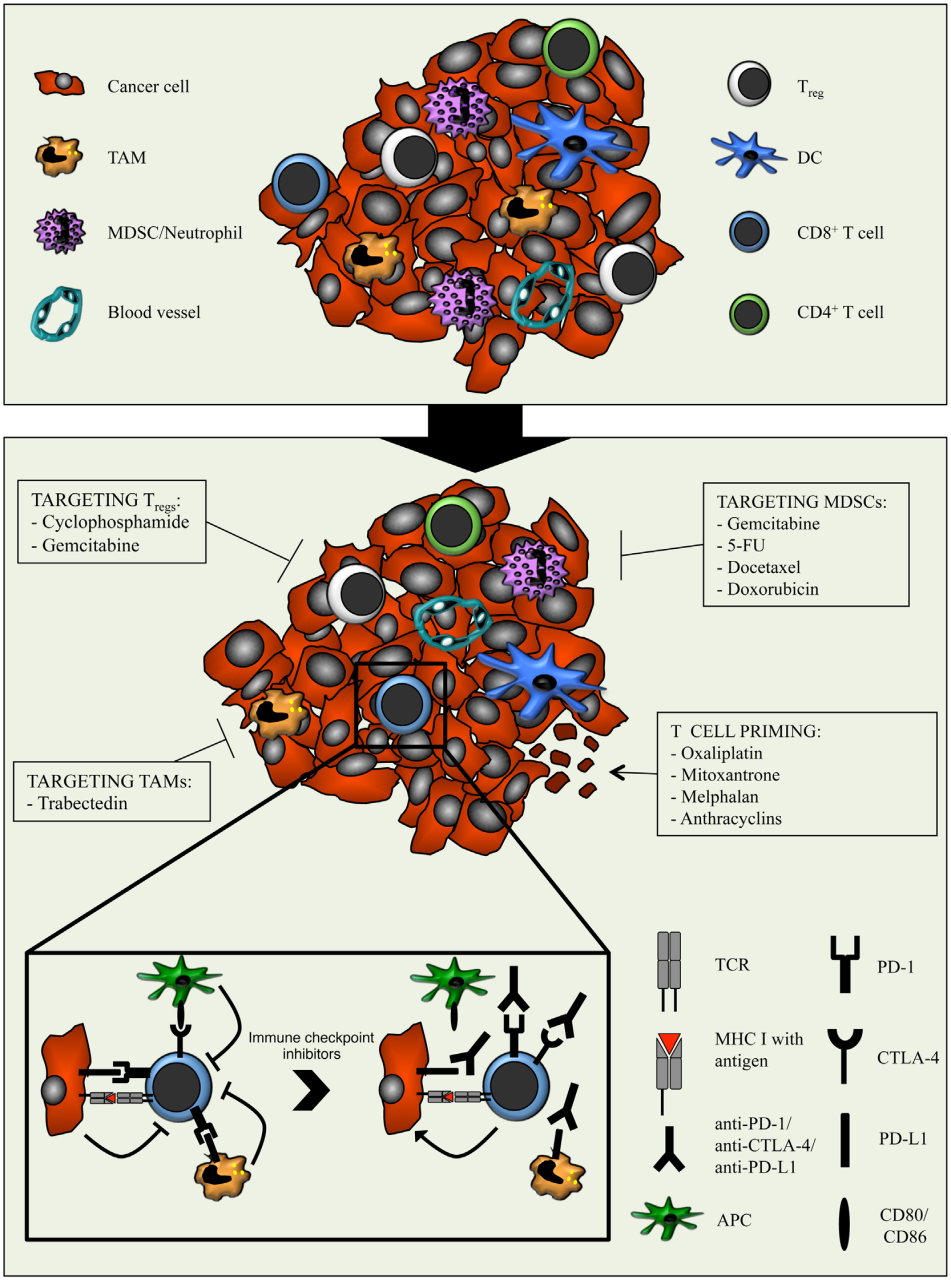
**Combination strategies aimed at relieving the immunosuppressive tumor microenvironment with chemotherapy and potentiating cytotoxic T cells with immune checkpoint inhibitors**. The tumor microenvironment is characterized by the presence of various immune cell types, including different subsets of adaptive immune cells and TAMs, MDSCs, and T_regs_. The latter dampens the anti-cancer activity of T cells through several mechanisms. Moreover, cancer cells and myeloid cells express PD-L1/PD-L2 and APCs express CD80/CD86. Binding of these molecules to PD-1 and CTLA-4 respectively, expressed on T cells, results in inhibitory signals that counteract T cell activation and function. The immunomodulatory properties of different types of chemotherapeutic drugs can be exploited to enhance anti-tumor immunity. By optimally matching the immunomodulatory features of specific chemotherapeutic drugs with the T cell-boosting effect of immune checkpoint inhibitors, the efficacy of immunotherapy might be improved.

Cancers do not merely consist of tumor cells, but comprise a variety of cell types that together form the tumor microenvironment (TME) (Figures 1 and 2). Infiltrating immune cells are of special interest because of their paradoxical role in cancer progression. While some immune cell populations have pro-tumorigenic properties, others counteract tumorigenesis (16–18). Many tumors are characterized by an immunosuppressive TME, which makes it unfavorable for anti-tumor immunity. To mount effective anti-tumor immunity, tumor-associated antigens need to be sampled and processed by antigen-presenting cells (APCs). After receiving specific maturation signals, these APCs migrate to tumor-draining lymphoid organs where antigens are presented to T cells. Upon activation and proliferation, tumor antigen-specific T cells migrate to the tumor bed where they exert their cytotoxic function. At every step of this T cell priming and effector process, tumors employ strategies to hamper anti-cancer immunity.

Tumors often show dysfunctional recruitment and activation of dendritic cells (DCs), which are the most potent APCs for initiating immune responses. Several studies show that tumor-infiltrating DCs display an immature phenotype (20, 21). Tumor-derived factors like IL10, IL6, CSF1, and VEGF interfere with DC maturation, causing failure to migrate to the tumor-draining lymphoid organs, and to provide the appropriate co-stimulatory signals required to stimulate T cells (21). Although a thorough analysis of the antigen-presenting myeloid immune cell compartment in the *MMTV-PyMT* mammary tumor model showed that intratumoral DCs are able to ingest and present tumor antigens to T cells, they fail to activate them (22). Nevertheless, even in these immunoevasive tumors, a rare population of IL12-expressing CD103^+^ DCs exists that is able to prime tumor antigen-specific T cells (23). Besides hampered T cell priming, the recruitment of activated T cells and their access into the tumor bed is often disrupted by the disorganized tumor vasculature and impaired expression of adhesion molecules on endothelial cells (24, 25). Some studies suggest that tumor-derived chemokines may cause selective trapping of T cells in the tumor stroma preventing access into the tumor bed (26). When tumor-specific T cells do succeed to reach the tumor, downregulation of MHC class I expression on tumor cells renders them invisible to T cell attack (27). Additionally, T cells face systemic and local tumor-induced immunosuppression, which limits their activation and function (28). Tumor-associated immunosuppression can be caused by tumor-infiltrating or systemically expanded myeloid cells or regulatory T cells (T_regs_) that – directly or indirectly via secretion of soluble mediators – hamper T cell priming and effector function or even induce T cell death (28). These mechanisms will be discussed in more detail later.

### Enhancing anti-tumor immunity by immune checkpoint inhibitors

To improve anti-tumor T cell immunity, different types of cancer immunotherapy approaches exist. While passive immunotherapy is based on adoptive transfer of (genetically engineered) autologous T cells, active immunotherapy boosts the endogenous immune response via cancer vaccines or inhibitors of immune checkpoints. The therapeutic effect of the latter is aimed at inhibition of negative immune regulatory pathways including cytotoxic T-lymphocyte-associated protein-4 (CTLA-4) and the programed cell death protein-1 (PD-1) receptor and one of its ligands, PD-L1 (B7-H1; CD274) (29). CTLA-4 is a member of the CD28 immunoglobulin superfamily and is expressed mainly on the surface of activated CD4^+^ T cells and T_regs_, while absent on naïve T cells (30). CTLA-4 plays a central role in maintaining immune tolerance by competing with CD28 to bind the ligands CD80 and CD86 present on activated APCs to inhibit T cell co-stimulation. The PD-1/PD-L1 axis shows similarities to that of CTLA-4. PD-1 is mainly expressed on activated T cells upon T cell receptor (TCR) engagement and on T_regs_, while naïve and memory T cells do not usually express this surface marker. Recent studies suggest that PD-1, rather than being a marker of activated T cells, identifies exhausted T cells (31). PD-L1 is expressed on multiple cell types, whereas expression of PD-L2 (B7-DC; CD273) seems to be restricted to APCs (32, 33). Like CTLA-4, binding of PD-L1/PD-L2 to its receptor results in an inhibitory signal that prevents T cell activation. While CTLA-4 blockade is hypothesized to act mainly in secondary lymphoid organs during the T cell priming phase, it is believed that blockade of PD-1 or PD-L1 targets the TME during the T cell effector phase (34). However, PD-1 can also play a role in the early T cell response as a regulator of CD8^+^ T cell expansion upon antigen recognition (35). In addition to its role in T cell priming, CTLA-4 also regulates the suppressive function of tumor-infiltrating T_regs_ (36, 37). In line with this, blockade of CTLA-4 in the B16 melanoma model acts locally in the TME by inactivating T_regs_ in an Fc-dependent manner resulting in a favorable shift in the effector T cell/T_reg_ ratio (38). The exact mechanisms of action of anti-CTLA-4 and anti-PD-1/PD-L1 are not completely clear. Just recently, the combination of anti-CTLA-4 and anti-PD-1 was reported to significantly increase the fraction of melanoma patients responding to immunotherapy compared to anti-CTLA-4 monotherapy-treated patients (39), emphasizing the different modes of action of CTLA-4 and PD-1.

The rational of using CTLA-4 blockade in cancer therapy is to release the brake on pre-existing tumor-reactive T cells and to generate new T cell responses. Ipilimumab (anti-CTLA-4) was the first immune checkpoint inhibitor that yielded a significant increase in survival of patients with metastatic melanoma, for which all conventional therapeutic options had failed (1). Interestingly, a broadening of the tumor-reactive T cell repertoire was reported upon ipilimumab treatment (40). In a second clinical study, ipilimumab was combined with dacarbazine in metastatic melanoma patients resulting in prolonged survival compared to dacarbazine alone (41). In both studies, a fraction of patients showed long-term durable responses (42). Similarly, clinical trials with anti-PD-1 have shown tumor regression in a substantial fraction of cancer patients (3). These initial results lead to an immense increase in clinical trials with drugs targeting the PD-1/PD-L1 axis in different cancer types, and many report anti-tumor efficacy (3–6, 43). Recent clinical observations show that the combination of anti-CTLA-4 and anti-PD-1 is more effective than either monotherapy (39). Although very successful and promising, a significant proportion of cancer patients do not show long-term benefit of immune checkpoint inhibitors. Therefore, it is of utmost importance to mechanistically understand intrinsic and acquired resistance to cancer immune checkpoint inhibitors, in order to identify biomarkers that can be used to pre-select those patients that will or will not benefit from cancer immunotherapy and to develop therapeutic strategies to overcome or bypass resistance mechanisms.

### What are the requirements for therapeutic response to checkpoint inhibitors?

To predict the response to immunotherapy per patient and tumor type, several variables should be taken into account. For successful activation of a T cell-mediated anti-tumor immune response, T cells need to “see” the cancer cells with their TCR. In general, there are three classes of tumor antigens that can potentially be recognized by T cells: viral antigens, self-antigens, and neo-antigens. Our T cell repertoire is basically built to recognize and respond to viral antigens because these antigens are perceived as foreign or non-self. However, only a subset of established human cancers expresses viral antigens. During the T cell maturation process, thymic selection eliminates maturing lymphocytes that display a high avidity for self-antigens. As a consequence, only low-avidity self-specific T cells can be found in the peripheral T cell repertoire, which may not be ideal for cancer immunotherapy. Non-synonymous somatic mutations can give rise to neo-antigens toward which no central T cell tolerance is present. Recently, neo-antigen-specific T cell responses have been reported in melanoma patients (44–46), indicating that these mutations can be recognized by T cells and induce tumor-specific T cell responses. In line with this, the number of predicted neo-antigens is linked with a metric for immune cytolytic activity based on gene expression in a large panel of cancer types (47). Thus, the extent of the mutational load of a certain tumor would serve – albeit at a low resolution – as a predictor of response to cancer immunotherapy. Indeed, a growing body of data supports this hypothesis (48). Whole-exome sequencing analyses revealed that melanoma and lung cancer – the two cancer types that show promising responses to immunotherapy – bear relatively high mutational loads compared to other types of cancer due to their exposure to DNA damaging insults like UV radiation and tobacco smoke, respectively (49). Recent studies uncovered that a high mutational load is associated with long-term clinical benefit to checkpoint inhibitors (8, 9). However, not all cancer patients with tumors bearing a high mutational load respond to checkpoint inhibitors, and some patients bearing tumors with low mutational load do (8, 9). Together, these results suggest that the mutational load of tumors is correlated with response to immune checkpoint inhibitors, but it cannot solely be used to predict response.

A growing body of clinical observations suggests that the intratumoral presence of pre-existing T cells is required for clinical benefit of immunotherapy (50). PD-1 expression on tumor-infiltrating CD8^+^ T cells has been suggested to identify the repertoire of clonally expanded tumor-reactive T cells (51). In addition, T cell infiltration correlates with PD-L1 expression in tumors and is associated with increased responsiveness to drugs targeting the PD-1/PD-L1 axis in melanoma patients (50, 52, 53). Expression of PD-L1 in tumors is one of the main characteristics pursued as a potential biomarker for response to PD-1/PD-L1 blockade. However, there are examples of tumors with high expression of PD-L1 that do not respond to PD-1 blockade, and PD-L1 negative tumors that do respond (53). Why certain tumors express PD-L1 and others do not remains to be elucidated.

Interestingly, expression of PD-L1 and responsiveness to immune checkpoint blockade is associated with genomic instability in different tumor types (54). Patients bearing mismatch-repair-deficient colorectal cancer (CRC) respond better to anti-PD-1 therapy than mismatch-repair-proficient CRC patients (54). In line with this, a microsatellite instable (MSI) subset of CRC patients shows high T cell influx (55). However, this is counterbalanced by simultaneous upregulation of checkpoint molecules including PD-1, PD-L1, and CTLA-4 leaving T cells dysfunctional (55). Moreover, in breast cancer, the expression of PD-L1 is correlated with TIL infiltration, and is mostly prevalent in basal-like, hormone-receptor-negative, and triple-negative tumors (56, 57). Furthermore, in glioma patients increased expression of PD-L1 in tumors was correlated with PTEN loss (58), suggesting that patients bearing genetically unstable cancer types might benefit from treatment with checkpoint inhibitors. Intriguingly, not only cancer cells, but also tumor-infiltrating myeloid cells express PD-L1, and counteract anti-tumor immunity in ovarian carcinoma and MSI-CRC (55, 59). Actually, PD-L1 expression on tumor-infiltrating immune cells has been suggested to be a better predictor of clinical response to anti-PD-L1 therapy than PD-L1 expression on cancer cells (52). It will be interesting to explore, which other cancer types are characterized by the influx of PD-L1-expressing myeloid cells.

In conclusion, to increase the efficacy of immunotherapy in different types of cancer, we could consider manipulating the many variables that determine intrinsic and acquired resistance. While altering cancer cell-intrinsic characteristics, such as mutational load or genomic instability, might be challenging, cancer cell-extrinsic characteristics, like an immunosuppressive TME, are easier to manipulate.

## Evasion from Cancer Immunotherapy: Relieving Immunosuppression as an Attractive Strategy to Improve the Efficacy of Immune Checkpoint Blockade

Established tumors are characterized by an abundant influx of a variety of immune cells with immunosuppressive activity, including T_regs_, myeloid-derived suppressor cells (MDSCs), and tumor-associated macrophages (TAMs) (Figures 1 and 2). There is accumulating evidence that interference with these immunosuppressive networks can improve anti-tumor immunity. Here, we discuss the different types of immunosuppressive immune cells present in the TME, and how blockade or reprograming of these cells or their downstream effects can enhance anti-tumor immunity and the efficacy of immune checkpoint blockade.

### Regulatory T cells

Regulatory T cells play an important role in maintaining homeostasis during infections and in preventing the development of autoimmune diseases by blocking proliferation and cytotoxic activity of effector T cells. The history of T_regs_ goes back to the 1970s, when it was discovered that a subpopulation of thymocytes induced tolerance to certain antigens in mice (60). A turning point in the research of these “suppressor cells” came in 1995. T_regs_, phenotyped as CD4^+^CD25^+^ cells, were shown to be important for self-tolerance in mice, as inoculation of CD4^+^ cells depleted of CD4^+^CD25^+^ cells resulted in autoimmunity in nude mice (61). Another big step forward in the characterization of T_regs_ was the identification of FOXP3, a member of the fork-head/winged-helix family of transcription factors and a key regulator of T_reg_ development and function (62). In the following years, the knowledge of T_regs_ expanded enormously. Two subpopulations of T_regs_ were identified: natural T_regs_ and induced T_regs_ (or adaptive T_regs_), which are formed in the thymus and in the periphery, respectively. Regardless of their origin, both natural and induced T_regs_ inhibit effector T cells (63).

In 1980, it was hypothesized that a T cell population in tumors suppresses anti-tumor immune responses (64). Indeed, many experimental studies support the notion that tumor-associated T_regs_ contribute to immune escape via suppression of anti-tumor CD8^+^ T cells. For example, elimination of T_regs_ in MO4 melanoma cell line-bearing mice results in T cell-dependent tumor rejection (65). Moreover, in a xenotransplant model for HER2^+^ ovarian cancer, adoptive transfer of autologous CD3^+^CD25^−^ T cells and DCs loaded with HER2^+^ antigen results in T cell-mediated tumor regression, whereas concomitant transfer of T_regs_ blocks this antigen-specific immune response (66). T_regs_ not only suppress CD8^+^ T cells, but also CD4^+^ T cells, NK, NKT, and B cells (67). T_regs_ exert their immunosuppressive function either by direct suppression of effector cells, or indirectly by affecting the activation state of APCs. Importantly, in order to exert their functions, T_regs_ need to be activated via their TCR, but once activated their suppressive function is non-specific (68, 69). The direct T cell-suppressive functions are mediated by release of cytokines, serine proteases and the expression of enzymes that catabolize ATP. For example, T_regs_ inhibit T cells via secretion of cytokines like TGFβ, IL10, and IL35 (70–72) or even induce T cell apoptosis by the release of granzyme B (GRZMB) or perforin (73–75). In addition, T_regs_ express CD39 and CD73, two ectoenzymes that generate the immunosuppressive molecule adenosine from extracellular ATP (76). It has been shown that T_regs_ from CD39 knock-out mice fail to inhibit CD4^+^CD25^−^ cell proliferation (76). Finally, CTLA-4^+^ T_regs_ can indirectly impair T cells by reducing the CD80/CD86 levels on APCs (36).

Supporting these data, increased numbers of intratumoral T_regs_ correlate with worse overall survival in patients with ovarian cancer, breast cancer, gastric cancer, and hepatocellular carcinoma (66, 77–81). Interestingly, this is not true for CRC in which a high number of CD8^+^ cells and FOXP3^+^ cells correlates with a good prognosis (82). This may be explained by the fact that T_regs_ in CRC attenuate inflammation against gut microbiota that would otherwise enhance tumor growth (82). These findings illustrate that the tumor context dictates the function of associated immune cells. Although strategies targeting CD25 (like the neutralizing monoclonal antibody daclizumab and the recombinant interleukin 2/diphtheria toxin conjugate Ontak) showed transient depletion of peripheral T_regs_ and increased activity of CD8^+^ T cells, these approaches only result in a modest clinical benefit in cancer patients (83, 84). This might be explained by the fact that CD25 is also expressed on active effector T cells, so the lack of specificity for T_regs_ might complicate their clinical applicability. Therefore, a mechanistic understanding of the role of T_regs_ in different tumor contexts will be important for the design of therapeutic strategies aimed at suppressing the downstream effects of T_regs_.

### Myeloid-derived suppressor cells

The first report describing the existence of MDSCs showed that bone marrow-derived cells were able to suppress the killing activity of splenocytes *in vitro* (85). These cells were called “natural suppressor cells” or “null cells” because they did not express markers of B, T, or NK cells or macrophages (86). Subsequently, these cells were found to expand in inflammatory conditions and in tumor-bearing hosts (85, 87). In tumor-bearing mice, tumor-derived growth factors trigger the accumulation of T cell suppressive myeloid cells in the bone marrow and spleen (87, 88). The identification of these cells was hampered by the lack of clear markers, which caused variation in terminology and ambiguity among researchers. In order to bring some clarity into the field, Gabrilovich and colleagues published a consensus paper in 2007 in which they coined the term “MDSC” to refer to a heterogeneous population of myeloid cells with the ability to suppress T cell activity (89). MDSCs consist of a group of immature and mature myeloid cells that are defined by their immunosuppressive function. Within the MDSC population, two subpopulations can be distinguished based on the expression of Ly6G and Ly6C: Ly6C^high^Ly6G^−^ monocytic-MDSC and Ly6C^low^Ly6G^+^ granulocytic-MDSC. In humans, MDSCs are defined as CD11b^+^CD33^+^HLA-DR^−^Lin^−^ cells with the addition of CD14 or CD15 to discriminate between monocytic- or granulocytic-MDSCs, respectively (90).

In patients with various cancer types, including melanoma, gastric, breast, and CRC, increased numbers of MDSCs in the circulation correlate with poor survival (91–93). Numerous cytokines have been implicated in the expansion of MDSCs during cancer progression, including G-CSF, GM-CSF, and stem-cell factor (SCF or KIT ligand) (94–96).

Myeloid-derived suppressor cells exert their immunosuppressive function by different mechanisms, one of which is the consumption of essential amino acids from the environment. MDSCs frequently express high levels of arginase I, which catabolizes arginine, thereby depriving T cells from arginine, which is essential for their metabolism and function (97, 98). l-Arginine is also the substrate of another enzyme highly expressed in MDSCs, called iNOS. The release of reactive oxygen species (ROS) and nitric oxide (NO) by iNOS can lead to the inhibition of MHC class II expression on APCs causing impaired antigen presentation to CD4^+^ T cells (99). Moreover, NO can cause apoptosis of CD8^+^ T cells (100). Another amino acid is tryptophan, whose breakdown by the enzyme IDO suppresses T cell proliferation. MDSCs isolated from human breast cancer tissues inhibit T cell proliferation and induce T cell apoptosis in an IDO-dependent manner (101). Moreover, IDO inhibitors enhance the therapeutic efficacy of anti-CTLA-4 treatment leading to intratumoral accumulation of T cells and improved survival in the B16 melanoma model (102). Additionally, the amino acid cysteine is also important for T cell activation and function. T cells depend on other cells (macrophages and DCs) for cysteine metabolism. MDSCs internalize cystine (formed of two cysteines linked via a disulfide bond), catabolize it to cysteine and, unlike macrophages and DCs, do not release it into the environment. Therefore, MDSCs limit the amount of cystine that macrophages and DCs can metabolize to activate T cells (103). Finally, MDSCs contribute to an immunosuppressive TME by inducing the development of T_regs_ in tumor-bearing mice, as adoptive transfer of MDSCs and CD4^+^ T cells in MCA26 colon carcinoma cell line-bearing irradiated mice, induces expression of FOXP3 in transferred T cells (104). Thus, these data suggest that MDSCs play an important role in creating an immunosuppressive network in tumors, supporting the idea that reprograming or depletion of MDSCs could benefit immunotherapy strategies. Strategies to inhibit MDSCs include blocking their development or recruitment, targeting their immunosuppressive molecules or depleting them.

### Tumor-induced neutrophils

In various cancer patients, a high neutrophil to T lymphocyte ratio in blood is associated with poor disease outcome (105, 106). Recent studies have reported that neutrophils also expand in experimental mouse tumor models, and that they exert immunosuppressive activity. A distinguishing feature of murine neutrophils is the expression of Ly6G, a surface marker shared with granulocytic-MDSC. When the T cell suppressive ability of neutrophils is confirmed, they can be categorized into the granulocytic-MDSC population (107). We recently showed in a mouse model for *de novo* breast cancer metastasis that neutrophils have a pro-metastatic phenotype and exert their function through suppression of CD8^+^ T cells. While depletion of Ly6G^+^ neutrophils results in decreased multi-organ metastasis, double depletion of neutrophils and CD8^+^ T cells reverses this phenotype (108). In line with this, chemotherapy-induced neutropenia correlates with improved overall survival in breast cancer patients (109). The metastasis-promoting role of neutrophils has also been demonstrated in UV-induced melanoma and in tumor inoculation models (110, 111). It would be interesting to study whether – as in the experimental tumor models – T cells in neutropenic cancer patients are more active. Interestingly, in 4T1-tumor-bearing mice, neutrophils inhibit the seeding of metastatic cells in the lung by the release of hydrogen peroxide (112). These data indicate a controversial role of neutrophils in metastasis that might be explained by the differences in tumor subtype or tumor model.

We and others have shown that T cell-suppressive neutrophils accumulate systemically during cancer progression in a G-CSF-dependent fashion (108, 113). In the transgenic *MMTV-PyMT* mammary tumor mouse model, tumor-derived G-CSF skews hematopoietic cell differentiation toward the granulocytic lineage in the bone marrow, resulting in increased numbers of immunosuppressive neutrophils in the circulation (113). In 4T1 mammary tumor-bearing mice, TGFβ polarizes mature neutrophils from cytotoxic anti-tumor activity toward pro-tumor immature immunosuppressive neutrophils (114). This is in line with previous findings identifying TGFβ as one of the drivers of pro-tumor polarized neutrophils (115). As such, it is tempting to speculate that for those tumors characterized by pro-metastatic neutrophils, inhibition of these cells – either by targeting upstream or downstream molecules – may be an interesting strategy for therapeutic intervention, in particular when combined with cancer immunotherapy.

### Tumor-associated macrophages

Macrophages are frequently the most predominant immune cell type in tumors. In the past, macrophages were subdivided into classically activated macrophages (M1) exerting microbicidal and anti-tumor activity, or alternatively activated macrophages (M2) exerting pro-tumoral, immunosuppressive, and tissue repair functions (116, 117). TAMs are frequently classified as M2 macrophages. However, there is a growing realization that this black and white distinction of macrophage subsets is too simplistic and does not accurately reflect the heterogeneity, plasticity, and versatility of macrophages (118). Transcriptome and bioinformatic analyses of cultured macrophages exposed to different stimuli revealed a spectrum of activation programs for each stimulus that goes beyond the M1 and M2 model (119). Based on these data, it is to be expected that TAMs will also change their phenotype and function according to the cytokine milieu present in a specific tumor type. In the vast majority of cancers, high intratumoral macrophage density correlates with poor prognosis (120, 121). However, macrophages in CRC are associated with good prognosis, and in other types of cancers, like prostate and lung cancer, their role is still controversial (122). Depletion of macrophages by genetic ablation of CSF-1 in the *MMTV-PyMT* mammary tumor model reduces metastasis formation without affecting primary tumor growth (123). Likewise, several other experimental studies have reported a pro-metastatic role of macrophages (124, 125). TAMs produce a variety of factors that foster tumor growth and invasiveness, angiogenesis, and immunosuppression (120, 124, 126).

Tumor-associated macrophages exert their immunosuppressive activity in a similar fashion as that of MDSCs. TAMs can express various enzymes like arginase 1, IDO (127–129), and cytokines like IL10 (130). Another mechanism by which TAMs suppress T cells is the upregulation of PD-L1. In hepatocellular carcinoma, high density of peritumoral macrophages that express PD-L1 correlates with worse overall survival (131). Co-culture experiments showed that PD-L1^+^ macrophages suppress T cell activity unless anti-PD-L1 antibody is added in the culture (131). Based on these immunosuppressive properties, it is tempting to speculate that interference with TAMs will unleash anti-tumor immunity. Indeed, this idea has recently been supported by experimental studies in mouse models for glioblastoma and pancreatic cancer showing that CSF-1/CSF-1R pathway blockade can shift TAM polarization toward an anti-tumor phenotype, resulting in enhanced CD8^+^ T cell-mediated anti-tumor immunity (132, 133). Similarly, targeting the CCL2/CCR2 chemokine pathway – involved in recruitment of monocytes and macrophages – relieves the immunosuppressive phenotype of TAMs and enhances anti-tumor CD8^+^ T cell responses (134, 135). Based on these encouraging results, clinical trials are ongoing in which compounds targeting TAMs are being tested in cancer patients. Preliminary results of a clinical trial with anti-CSF-1R in patients with various types of solid malignancies showed a decrease in TAMs and an increase in intratumoral CD8/CD4 ratio (10).

### Blocking the suppressors to release anti-tumor T cells

As discussed above, many immunosuppressive cells and mediators can be identified in the TME that dampen anti-tumor T cell responses and may contribute to immune escape upon cancer immunotherapy. The combination of compounds that relieve immunosuppression with T cell-boosting therapy seems attractive to overcome immune tolerance toward the tumor.

Regulatory T cells seem to be interesting targets, since, as discussed earlier in this review, these cells suppress the functionality of CD4^+^ and CD8^+^ effector cells. In line with this, in the transgenic TRAMP prostate cancer model – engineered to express prostate-specific antigen (PSA) – T_reg_-depletion enhances IFNγ production by PSA-specific CD8^+^ T cells (136). This augmented effect of anti-tumor immunity is further enhanced by CTLA-4 blockade, and results in delayed tumor growth. Interestingly, the same experiments performed in the parental TRAMP model show only a modest activation of PSA-specific T cells upon anti-CD25 and anti-CTLA-4, and no survival benefit, suggesting the requirement of a tumor-specific antigen for this anti-tumor response (136). In the ID8 ovarian cancer model, tumor-infiltrating T_regs_ – which express both CTLA-4 and PD-1 – are reduced upon CTLA-4 and PD-1 dual blockade coinciding with increased tumor-infiltrating CD8^+^ T cells (137). However, additional depletion of T_regs_ does not further enhance this effect. In the same model, blockade of PD-L1, expressed on tumor cells and tumor-infiltrating immune cells, reduces the number of MDSCs and T_regs_ and enhances the frequency of effector T cells, resulting in prolonged survival (138). Furthermore, in a mouse model for rhabdomyosarcoma, PD-1 blockade increases the number of tumor-infiltrating CD8^+^ T cells, but does not change their activation status. Upon interference with the chemokine receptor CXCR2, which prevents MDSC trafficking into the tumor, enhanced activation of CD8^+^ T cells is observed (139). Blockade of CXCR2 improves the therapeutic efficacy of anti-PD-1 treatment resulting in a significant survival benefit (139). Moreover, in a mouse model of pancreatic ductal adenocarcinoma, blockade of CSF-1/CSF-1R signaling results in macrophage reprograming to support anti-tumor immune function and modestly delays tumor growth (133). TAMs obtained from anti-CSF1 treated mice are impaired in suppressing CD8^+^ T cell proliferation compared to control TAMs. The induction of CTLA-4 expression on CD8^+^ T cells and PD-L1 expression on tumor cells suggests the onset of acquired resistance to effective anti-tumor immune responses. Combining anti-CTLA-4 and anti-PD-1 with a CSF-1R inhibitor shows profound synergy with a significant reduction in tumor burden (133). Thus, together these results indicate that alleviation of immunosuppression reactivates anti-tumor immunity, which can be further enhanced by checkpoint inhibition.

## Immunomodulatory Properties of Chemotherapeutic Drugs

Although various novel compounds targeting tumor-associated myeloid cells and their immunosuppressive mediators are being developed and tested, their clinical availability is still limited. An alternative and clinically available strategy is to relieve immunosuppression by exploiting the immunomodulatory effects of conventional anti-cancer strategies like chemotherapy (Figure 2). The impact of chemotherapeutic drugs on the proportion and phenotypic and functional characteristics of immune cells is to a great extent dictated by the type of drug and the dosing scheme: while high-dose chemotherapy usually results in lympho- or myelodepletion, low-dose (metronomic) treatment has more subtle anti-angiogenic and immunomodulatory effects (140, 141). In this section, we discuss the effects of chemotherapy on the immunosuppressive TME.

### The impact of chemotherapy on T cell priming

Optimal T cell priming is dependent on antigen processing, presentation, and co-stimulation by properly matured and activated DCs. As discussed, impaired DC function and T cell priming are important mechanisms of immune escape by tumors. Certain chemotherapeutics induce anti-cancer immune responses by improving the recruitment and functionality of intratumoral DCs (142, 143). For example, low-dose cyclophosphamide promotes DC maturation (144). Besides the enhanced release of tumor antigens through induction of cancer cell death, chemotherapeutics, including oxaliplatin, doxorubicin, mitoxantrone, and melphalan, induce HMGB1 release and calreticulin translocation in cancer cells, facilitating antigen uptake by DCs and subsequent T cell stimulation (145–147). In addition, in the MCA205 fibrosarcoma model, anthracyclins induce the differentiation of myeloid cells in the tumor bed toward a DC-like phenotype in an ATP-dependent manner (142). In these relatively high immunogenic tumor models, the activated T cells subsequently enhance the anti-cancer efficacy of chemotherapy (142, 143, 145).

In less immunogenic models, such as *de novo* tumorigenesis models, an important role for T cells in chemotherapy efficacy is lacking (120, 148, 149). One possible explanation is that spontaneously arising tumors are characterized by local and systemic immunosuppression, which may overrule any chemotherapy-induced T cell responses. Indeed, in the *MMTV-PyMT* mammary tumor model, TAM-derived IL10 indirectly blocks anti-tumor CD8^+^ T cell activity by suppressing IL12 expression by intratumoral DCs upon paclitaxel treatment (149). These results apply to human breast cancer patients since low CD68^+^ macrophage over CD8^+^ T cell ratio prior to neo-adjuvant chemotherapy correlates with a better pathologic response (120). Moreover, high levels of *IL12A* mRNA in human breast cancer samples correlates with expression of DC-related transcription factors and *GRZMB*, *CD8A*, and *IFN*γ expression, suggesting an active anti-tumor T cell response (149). However, the role of TAMs and their potential suppressive function in cancer patients was not evaluated. Together, these results suggest that therapeutic targeting of TAMs could enhance the functionality of intratumoral DCs and anti-tumor T cell responses in chemotherapy treatment.

### Impact of chemotherapy on T_regs_

With the knowledge that T_regs_ play an important role in suppressing effector T cell responses, a lot of effort has been put into the identification of chemotherapeutic drugs that target these cells. The best studied is cyclophosphamide, an alkylating agent, which crosslinks DNA, thus interfering with replication. Cyclophosphamide is known for its dose-dependent effect on the immune system. High doses of cyclophosphamide result in immunosuppression by reducing T cell proliferation and inducing apoptosis, thus making it useful for the prevention of graft-versus-host disease or rejection of transplanted organs (150, 151). In contrast, low doses selectively ablate T_regs_ and dampen their T cell suppressive ability (152). While the anti-tumor effect of high-dose cyclophosphamide is mainly due to its cytotoxic activity against cancer cells, the anti-tumor effect of low-dose cyclophosphamide depends on its immune-modulatory effects (153). Indeed, studies in T cell-deficient mice bearing inoculated tumors show loss of the anti-cancer activity of low-dose cyclophosphamide (153, 154). Moreover, reinfusion of CD4^+^CD25^+^ T cells in tumor-bearing mice, pre-treated with low-dose cyclophosphamide, abrogated the anti-tumor effect of the drug, emphasizing that T_regs_ counteract the therapeutic efficacy of the drug (153). In line with this, patients with different types of metastasized solid tumors receiving low-dose metronomic cyclophosphamide show a specific decrease of T_regs_ in the periphery with concomitant enhancement of NK lytic activity and T cell proliferation (155). In cancer patients receiving higher doses of metronomic cyclophosphamide, all lymphocyte populations were depleted, emphasizing the importance of accurate drug dosing to achieve selective T_reg_ depletion (155). It has been proposed that the increased sensitivity of T_regs_ for cyclophosphamide is linked to their low ATP levels. Low levels of ATP result in decreased synthesis of glutathione, which is important for cyclophosphamide detoxification (156).

Another chemotherapeutic drug affecting T_regs_ is gemcitabine, a nucleoside analog interfering with DNA replication. In an orthotopic pancreatic cancer model, gemcitabine reduces the percentage of T_regs_ in the tumor resulting in a small but significant survival benefit (157). Whether this also results in improved CD8^+^ and CD4^+^ T cell activity remains unknown. A study performed in cancer patients showed that the percentage of T_regs_ in blood was decreased after gemcitabine treatment (158). Among the CD4^+^ cells, T_regs_ were identified as the most proliferative cells, which may explain the selectivity of gemcitabine for these cells. However, the effect of gemcitabine on other T cell populations was not assessed in this study (158). Also, other (combinations of) chemotherapy drugs have been reported to influence the presence or function of T_regs_ (159, 160).

### Chemotherapeutics with inhibitory activity toward tumor-associated myeloid cells

Several chemotherapy drugs have been implicated in the selective reduction of MDSCs in the tumor and spleen of tumor-bearing mice (161, 162). In an EL4 inoculation tumor model, a set of chemotherapy drugs was tested for their influence on the number of splenic and intratumoral MDSCs (161). This study showed that high-dose gemcitabine and 5-fluorouracil (5-FU), two anti-metabolite drugs that interfere with DNA replication, reduce MDSC accumulation (161). Consequently, 5-FU-mediated MDSC depletion results in increased IFNγ-producing intratumoral CD8^+^ T cells. This effect is reverted by adoptive transfer of MDSCs, suggesting that the effect of 5-FU is exerted through MDSCs (161). Similar results were obtained in the MCA203 cell line inoculation sarcoma model combined with cytotoxic T cell transfer (163), highlighting the critical role of MDSCs in dampening T cell activity upon 5-FU treatment. While the exact mechanisms underlying the selectivity of 5-FU for MDSCs are unknown, it has been proposed that 5-FU inhibits the enzyme thymidylate synthase and that the resistance to 5-FU is due to insufficient inhibition of this enzyme (164). Indeed, low levels of thymidylate synthase are found in MDSCs compared to splenocytes and EL4 tumor cells, suggesting that 5-FU selectivity for MDSCs could be due to this low enzymatic expression (161).

High-dose gemcitabine induces similar effects on MDSCs as 5-FU (162). *In vitro* analyses of splenocytes from TC-1 lung cancer-bearing mice showed the cytotoxic specificity of gemcitabine for MDSCs, while CD4^+^, CD8^+^ T cells, and B cells are unaffected (162). Although the exact mechanism underlying this specificity has not been identified, it has been hypothesized that gemcitabine induces apoptosis in MDSCs (162). Yet, a thorough mechanistic analysis of gemcitabine-induced apoptotic cell death in various immune cell populations has not been performed. In the 4T1 breast cancer mouse model, gemcitabine treatment also reduces splenic MDSC accumulation, which results in increased proliferation and IFNγ production by splenic lymphocytes upon antigen stimulation compared to untreated mice (165). However, no difference in anti-cancer efficacy of gemcitabine was observed between immunocompetent and nude mice, indicating a T cell-independent mechanism of 4T1 tumor control by gemcitabine (165). Perhaps, this observation might be explained by the presence of other immunosuppressive cells in the TME, like T_regs_ or macrophages.

The beneficial effect of chemotherapeutic drugs on the immunosuppressive TME is not only a direct result of reduced MDSC numbers, but also a result of a more favorable phenotype of the remaining MDSCs. For example, in the 4T1-Neu mammary tumor model, docetaxel reduces splenic granulocytic-MDSCs and enhances CD8^+^ and CD4^+^ cytotoxic activity (166). The remaining MDSCs exhibit a different phenotypic profile compared to MDSCs from untreated mice. In line with these *in vivo* findings, MDSCs pre-treated with docetaxel induce the proliferation of OVA-exposed OT-II CD4^+^ T cells compared to untreated MDSCs *in vitro*, suggesting that docetaxel treatment induces a phenotypical switch to a more favorable state (166). Likewise, doxorubicin selectively decreases the proportion of MDSCs in the 4T1 breast tumor model via apoptosis and subdues the immunosuppressive phenotype of the remaining MDSCs. The remaining MDSCs have a lower expression of immunosuppressive molecules like ROS, ARG-1, and IDO (167). This less suppressive environment caused by doxorubicin enhanced the activity of adoptively transferred T helper cells (167). Interestingly, some subpopulations of MDSCs may be more susceptible to chemotherapy than others. Whether chemotherapy selectively depletes pro-tumorigenic MDSCs or skews them toward an anti-tumor phenotype is unknown. Future studies using lineage tracing methodologies would provide more insight into this topic.

Besides the favorable immunomodulatory “off-target” effects of various chemotherapeutic drugs, these drugs can at the same time exert less desirable functions. For instance, in addition to its inhibitory effect on T_regs_, cyclophosphamide increases the number of CD11b^+^Gr1^+^ MDSCs. In a transgenic mouse model for melanoma, a single injection of low-dose cyclophosphamide increases the accumulation of MDSCs in the tumor and spleen, stimulates their immunosuppressive ability by inducing NO and ROS production, and reduces splenocyte proliferation (168). In line with these findings, MDSCs accumulate in the blood of breast cancer patients after treatment with doxorubicin or cyclophosphamide (169). This may be due to IFNγ release by CD4^+^ and CD8^+^ T cells that promotes survival of MDSCs (170). Based on these data, a combination of cyclophosphamide and cancer immunotherapy might not work; however, additional studies in other tumor models should be performed to test this.

Another study underscoring the complex impact of chemotherapy on myeloid cells shows that in EL4-tumor-bearing mice 5-FU induces IL1β secretion in MDSCs in an Nlrp3 inflammasome-dependent manner (171). Using depletion experiments and knock-out mice, it was shown that the MDSC-derived IL1β triggers IL17 production by CD4^+^ T cells, which limits the anti-cancer efficacy of 5-FU (171). These data highlight that the effect of certain chemotherapy drugs is not simply limited to depletion of immunosuppressive cells but these drugs also change the functionality of cells that may impair their efficacy. These results suggest that the combination of chemotherapeutic and immunomodulatory compounds must be chosen carefully to increase their anti-cancer efficacy (172).

While several chemotherapy drugs have been reported to target MDSCs, thus far only one drug seems to strongly affect TAMs. Trabectedin, a drug that binds DNA and affects transcription and DNA repair pathways, depletes macrophages, and suppresses the differentiation of monocytes in the tumor bed in the transplantable MN/MCA1 fibrosarcoma tumor model through a TRAIL-dependent mechanism (173). Importantly, this macrophage selectivity is also observed in sarcoma patients after trabectedin neo-adjuvant treatment (173). It would be interesting to assess whether the anti-cancer activity of trabectedin is CD8^+^ T cell mediated. The macrophage-depleting effect of trabectedin makes it an interesting candidate for combination strategies with immunotherapy.

As discussed before, many studies illustrate the complexity of immunomodulation by conventional chemotherapeutics, which is highly context-dependent. The differential effect on specific immune cells of different types of chemotherapeutics is to a large extent dependent on the timing and dosing schedule. While high-dose chemotherapy often depletes immune cell subsets, low-dose metronomic chemotherapy exerts a more subtle anti-angiogenic and immunomodulatory mode of action (140, 141). It will be interesting to perform a side-by-side comparison of various types of chemotherapies administered at high versus low (metronomic) dose and evaluate their immunomodulatory effects, followed by more mechanistic studies. Ideally, these types of experiments would be performed in clinically relevant mouse models that faithfully recapitulate human cancer (Box 1) to facilitate clinical translation.

Box 1 **Experimental mouse models to study the anti-tumor immune response.**

Understanding the complex crosstalk between innate and adaptive immune cells and (disseminated) cancer cells requires the use of preclinical mouse models that faithfully recapitulate human cancer. The most widely used experimental mouse models are carcinogen-induced cancer models and cell line inoculation models. The latter is based on inoculation of large numbers of (genetically modified) homogenous cancer cells grown in 2D conditions. Implantation of these cells often results in massive cell death, thereby priming an effective anti-tumor immune response. Shaping of the tumor immune microenvironment during cancer progression in these models can hardly take place in the short amount of time that it takes for transplanted tumors to grow to their maximum tolerated size. Of notice, when implanting human cancer cells, either patient-derived tumor material or established human cancer cell lines, immunocompromised mice are used, thereby excluding the important role of the adaptive immune system.

While cell line inoculation models proved useful to decipher some aspects of the anti-tumor immune response, we should keep in mind that these models do not reflect physiological processes as they occur in human patients. Genetically engineered mouse (GEM) models, which develop *de novo* cancers, generally mimic human cancer genetically – because of the introduction of specific driver mutations – and histopathologically (180). In addition, tumor progression occurs in a multi-step nature in their natural microenvironment shaping the local immune responses (Figure 1), therefore mimicking the human setting. In contrast to inoculation models expressing known tumor antigens, the anti-tumor immune response in GEM models can be considered a black box. Due to their cellular and genetic heterogeneity, GEM models induce a variety of T cell responses directed against multiple unknown tumor neo-antigens, which faithfully reflects human cancer. Interestingly, comparative studies have shown that inoculation models greatly differ from GEM models in terms of response to anti-cancer therapies and endogenous T cell responses (181, 182). The advantages and disadvantages of different experimental mouse models in studying responsiveness to anti-cancer therapy have been recently discussed (14, 183).

## Future Perspectives: Exploiting the Immunomodulatory Properties of Chemotherapeutic Drugs to Improve Cancer Immunotherapy

Given their immunomodulatory properties, conventional chemotherapy drugs are interesting candidates to combine with T cell-boosting immunotherapy – a concept termed chemo-immunotherapy (174). Clinical trials report enhanced anti-tumor T cell responses in cancer patients treated with chemotherapy in combination with cancer vaccines (13). Moreover, clinical testing of chemotherapy combined with other immunotherapy approaches like adoptive transfer of (genetically engineered) autologous T cells or toll-like receptor (TLR) agonists are likely to be explored in the near future. Indeed, various experimental studies support the concept that chemotherapy-induced relief of immunosuppression could improve cancer immunotherapy. In a passive immunotherapy setting, in the MC203 fibrosarcoma and TC-1 lung cancer cell line inoculation models, low-dose gemcitabine and 5-FU reduced the splenic population of CD11b^+^Gr1^+^ MDSCs, resulting in enhanced anti-tumor activity of adoptively transferred tumor-specific CTL (163).

The results obtained in preclinical models combining chemotherapeutics with immune checkpoint inhibitors are promising. The immunomodulatory effects of melphalan – administered in a subtherapeutic dose – synergizes with CTLA-4 blockade in a plasmacytoma model (175). *In vitro* assays revealed that splenocytes obtained from melphalan-treated mice co-cultured with anti-CTLA-4 induced tumor cell cytotoxicity, while splenocytes from non-treated mice – irrespective of CTLA-4 blockade – did not (175). Furthermore, in the poorly immunogenic AB-1 malignant mesothelioma and Lewis lung cancer (LLC) inoculation tumor models, a combination therapy of gemcitabine and CTLA-4 blockade synergizes, inducing potent anti-tumor immune responses and subsequent regression of tumors in a CD4- and CD8-dependent manner (176). In addition, in a subcutaneous murine mesothelioma model, synergy is observed between cisplatin and CTLA-4 blockade, resulting in a profound anti-tumor effect that is characterized by increased influx and activation of CD4^+^ and CD8^+^ T cells in the tumor (177). Moreover, preclinical studies in mice show that doxorubicin, cisplatin, and paclitaxel in addition to their immunomodulatory role, can sensitize tumor cells for CTL attack in a direct manner (178). Here, chemotherapy causes increased permeability of tumor cell membranes to GRZMB, which sensitizes cancer cells to the cytotoxic effects of T cells and improved different cancer immunotherapy strategies (178). Together, these preclinical studies – albeit limited numbers – show the potential to exploit immunomodulatory chemotherapeutic drugs to improve the efficacy of checkpoint blockade.

Clinical trials that evaluate the combination of chemotherapeutic drugs and checkpoint inhibitors in cancer patients are still limited. Some studies in melanoma and lung cancer have used chemotherapeutics in combination with checkpoint blockade resulting in improved survival compared to chemotherapy alone (41, 179). However, the rational of these studies was not to evaluate the effect of treatment on the immunosuppressive microenvironment. Moreover, the design of clinical trials makes it impossible to perform a structural comparison in patients to study the effect of the immunosuppressive microenvironment on immunotherapy efficacy and whether this efficacy can be enhanced by adding chemotherapeutics to the treatment regimen. Therefore, we need to rely on preclinical research in mouse tumor models that faithfully recapitulate human cancer in terms of the genetic composition, anti-tumor immunity, and the immunosuppressive TME (Box 1). Results obtained in mouse models that mimic human cancer might shape the design of clinical trials and guide toward interesting treatment strategies. There are still various important questions that need to be addressed to maximally exploit the therapeutic efficacy of chemotherapy and immunotherapy combinations, like the determination of the most optimal combinations. Based on preclinical findings, different cancer types will likely require different combinations of therapy. In addition, despite the devastating effects of metastatic disease, mechanistic insights into the site-specific therapeutic response profiles and resistance mechanisms of cancer immunotherapy are completely lacking. Moreover, it is critical to gain insights into the mechanisms underlying intrinsic and acquired resistance to cancer immunotherapy. To answer these questions within the next decade, it is critical that basic researchers and clinicians intensify their efforts to join forces, so that results from preclinical research can guide the design of clinical trials, and the results from clinical trials, in turn, can guide mechanistic studies in mouse models. Together, these efforts will improve treatment strategies using chemotherapeutics to alleviate immunosuppression and enhance cancer immunotherapy.

## Author Contributions

KK, CS, and KV reviewed relevant literature and drafted the manuscript. KV revised the manuscript and supervised KK and CS. All authors read and approved the final manuscript.

## Conflict of Interest Statement

The authors declare that the research was conducted in the absence of any commercial or financial relationships that could be construed as a potential conflict of interest.
